# Electrochemical Modulation of Laser‐Induced Covalent Functionalization of Graphene

**DOI:** 10.1002/advs.75454

**Published:** 2026-04-27

**Authors:** Tamara Nagel, Linda Feuerstein, Kevin Gerein, Stefan Wolff, Janina Maultzsch, Andreas Hirsch, Inez M. Weidinger, Frank Hauke

**Affiliations:** ^1^ Department of Chemistry and Pharmacy & Center of Advanced Materials and Processes (ZMP) Friedrich‐Alexander Universität Erlangen‐Nürnberg Fürth Germany; ^2^ Chair of Electrochemistry Technische Universität Dresden Dresden Germany; ^3^ Department of Physics Chair of Experimental Physics Friedrich‐Alexander‐Universität Erlangen‐Nürnberg Erlangen Germany

**Keywords:** electrochemistry, functionalization, graphene, photochemistry, raman spectroscopy

## Abstract

The spatially controlled covalent functionalization of graphene allows for local modulation of its electronic and chemical properties. However, existing approaches either offer high lateral precision without independent reaction tuning or provide energetic control at the expense of spatial confinement. Herein, we introduce a photoelectrochemical‐driven strategy that combines the spatially resolved laser activation of precursors with electrochemical reaction gating that allows for modulation of the electron‐transfer driving force. Within a defined potential window, the degree of covalent addend binding on graphene can be continuously tuned by varying the applied bias under otherwise identical irradiation conditions. The electrochemical potential serves as an independent control parameter, complementing laser power and irradiation time, and thus provides an additional tool for the calibration of the degree of functionalization. Negative potentials enhance the covalent addend binding, whereas positive potentials suppress the reaction. This allows for a reproducible switching between active and inactive states. The electrochemically defined “off” condition enables in situ Raman characterization in the presence of the precursor solution without inducing unintended additional addend binding, enabling uninterrupted, stepwise monitoring of the functionalization process. This combination of laser‐pathway guided spatially resolved patterning and voltage‐controlled reaction tuning establishes a dual‐control platform for future precision graphene chemistry.

## Graphical abstract

Photoelectrochemical functionalization of graphene introduces the electrochemical potential as an additional parameter for the laser‐induced covalent modification of graphene. Tuning of the potential allows for amplification or suppression of the functionalization reaction with constant laser parameters, which enables in situ Raman analysis of functionalized areas without causing unintended alterations by reversibly suppressing the reaction.

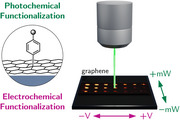

## 1 Introduction

The development of tailored materials with spatially defined properties has become a central objective in the 2D materials community. Site‐selective, covalent functionalization of graphene represents a key strategy to locally modulate its intrinsic properties by binding functional addends with lateral precision in clearly defined, adjacent areas [1, 2, 3]. Over the past years, a variety of approaches have been developed to achieve such spatial control, either by introducing pre‐structuring steps prior to the functionalization reaction [4, 5, 6, 7, 8, 9, 10] or by employing a local activation stimulus that can be moved in free pathways across the material to initiate covalent functional group binding at selected positions [11, 12, 13]. Common activation sources include plasma [13] or laser irradiation [11, 12] as well as locally applied electrochemical potentials, for instance in scanning electrochemical cell microscopy [14]. Among these, laser‐induced activation of precursor molecules has received particular attention, as it enables the direct laser “writing” of functional addends onto the graphene lattice [15, 16, 17, 18, 19, 20, 21, 22]. In recent years, extensive studies of this laser “writing” process have provided detailed insights into the influence of the relevant parameters and revealed that for some molecules the underlying reaction mechanism is governed by a hot‐electron transfer from graphene to the reactant, thereby enabling highly precise, laser‐pathway‐guided functionalization [17].

Next to the photochemical activation during the laser “writing” process, electron transfer can also be initiated electrochemically by shifting the Fermi level of graphene above the lowest unoccupied molecular orbital (LUMO) of the precursor molecule via an externally applied potential. This method has been extensively investigated on various carbon surfaces using diazonium compounds and, more recently, has also been demonstrated with iodonium reagents [14, 23, 24, 25, 26, 27, 28]. Diaryliodonium precursors are generally less reactive than diazonium compounds, which allows for better control over the reaction kinetics using electrochemical parameters such as the applied electrochemical potential. However, in this case, the spatial resolution is limited by the contact area between electrically connected graphene and the precursor solution, generally resulting in larger functionalized regions compared to the laser “writing” concept. This limitation in lateral control over addend binding in wet‐chemical approaches was recently addressed through the development of a solution‐based laser “writing” technique, which combines the availability of molecular precursors in solution with locally targeted activation. Using this approach, an effectively unlimited supply of precursors is provided through continuous diffusion to the graphene‐solution interface, enabling exceptionally high degrees of functionalization [29]. One major drawback of the established laser‐induced‐functionalization approaches is further unintended side‐functionalization during characterization (“reading”) that needs laser irradiation, such as Raman spectroscopy. This unintended functional addend binding can be prevented by removal or irreversible deactivation of the reactant after the “writing” step, which renders an in situ characterization of stepwise processes impossible. In addition to this, recent investigations of laser “writing” with high photon energy and/or laser power revealed the appearance of unintended side‐functionalization in adjacent areas [17]. This restricts the lateral control of the laser‐induced functionalization of graphene when aiming for particularly high degrees of functionalization.

In this work, we combined for the first time the photochemical (laser “writing”) and electrochemical strategies for the covalent functionalization of graphene, using *bis*(4‐fluorophenyl)iodonium triflate as a model precursor compound. This photoelectrochemical concept aims to exploit the complementary advantages of both methods to achieve high‐precision functionalization. While the laser “writing” pathway enables localized spatial patterning, the electrochemical addressability of graphene provides continuous control over its electronic properties, thereby influencing the electron‐transfer kinetics. We investigated the influence of the applied electrochemical potential on the degree of functionalization, as quantified by Raman spectroscopy. Moreover, the laser “writing” process was switched between “on” and “off” regimes by adjusting the applied potential. Finally, Raman mapping was performed under “off” conditions to characterize the functionalized graphene areas in situ, without inducing additional functionalization by Raman laser irradiation. This approach provides the unprecedented opportunity to in situ monitor the stepwise covalent addend binding process without the need for additional experimental operations. In contrast to purely photochemical approaches, which rely exclusively on irradiation parameters to control reactivity, and electrochemical methods, which lack spatial selectivity, the present strategy introduces an additional electrochemical control dimension that enables independent tuning of the electron‐transfer kinetics under constant irradiation conditions. This allows continuous modulation of the electron‐transfer driving force during spatially resolved covalent functionalization, thereby decoupling spatial control from reaction energetics and providing a new level of tunability in the patterned functionalization of graphene.

## 2 Results and Discussion

**FIGURE 1:**
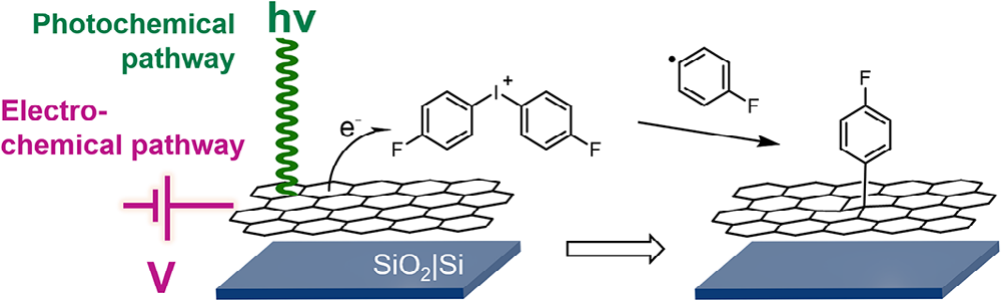
Schematic representation of the proposed photoelectrochemical functionalization mechanism: Electrochemical modulation of the graphene Fermi level in combination with laser excitation enables electron transfer from graphene to the precursor molecule, intermediate radical formation, and subsequent covalent attachment of the *p*‐fluorophenyl substituent to the graphene lattice.

A schematic representation of the proposed photoelectrochemical functionalization of graphene is depicted in Figure 1. As an initial step, the electrochemical modification of graphene with *bis*(4‐fluorophenyl)iodonium triflate was investigated in the absence of laser irradiation to find a suitable potential window for the combined approach, where a purely electrochemically‐induced binding of functional addends can be excluded. Graphene, generated by chemical vapor deposition (CVD), was deposited on a Si/SiO_2_ substrate using a wet transfer technique (see Section S1.2). The graphene‐covered substrate was subsequently mounted in a specially designed electrochemical cell, with the graphene sheet serving as the working electrode (WE) (see Figure S1 for a detailed view of the experimental setup).

**FIGURE 2:**
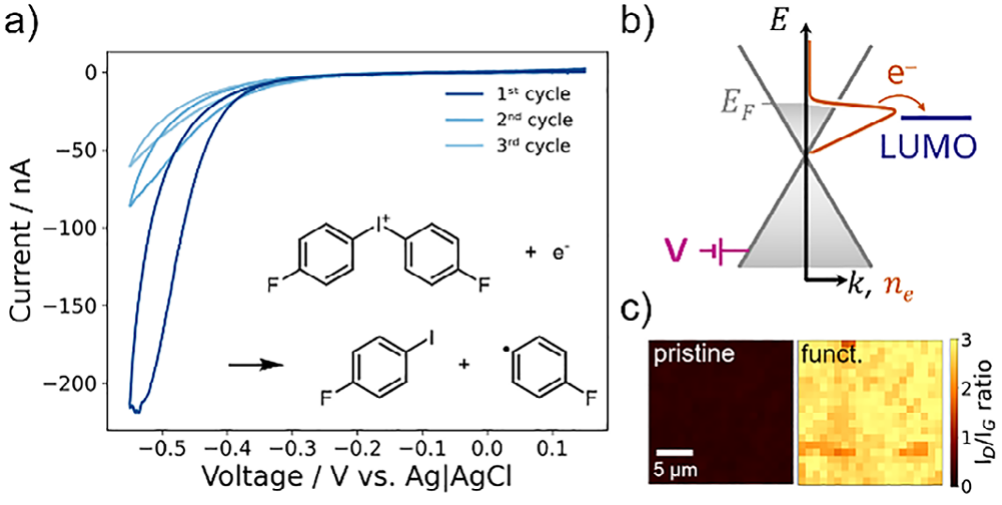
(a) Cyclic voltammogram (CV) of bis(4‐fluorophenyl)iodonium triflate (1 mm) in aqueous KCl electrolyte solution (0.1 m) at a scan rate of 1 mV s^−1^. (b) Schematic illustration of heterogeneous electron transfer from graphene to the lowest unoccupied molecular orbital (LUMO) of the reactant molecule induced by electrochemical modulation of the Fermi level *E_F_* via an external voltage V (the orange line represents the electron distribution *n_e_* in graphene above the Dirac point). (c) Raman *I_D_/I_G_* ratio map of pristine graphene and after electrochemical functionalization.

The electrochemical modification of carbon surfaces (also referred to as “electrografting”) is a well‐known method for large‐scale surface modification and was previously reported for carbon surfaces using diaryl iodonium cations as reactant [25, 27]. The cyclic voltammogram (CV) obtained for bis(4‐fluorophenyl)iodonium (1 mm in aqueous 0.1 m KCl solution, see Figure 2a) shows the characteristic shape of an electrochemical surface functionalization via a radical mechanism. Here, a functionalization reaction appears starting from a lowering of the applied potential below the onset potential to –0.35 V vs. Ag|AgCl, induced by a heterogeneous electron transfer from the graphene to the reactant, facilitated by a shift of the Fermi level of graphene above the LUMO of the reactant (scheme depicted in Figure 2b). A corresponding increase in the cathodic current is recorded in the first cycle of the CV. The so‐formed radical compound then decomposes, forming a phenyl radical, which covalently binds to the graphene surface. A cathodic peak at −0.53 V vs. Ag|AgCl may arise from mass transport limitations associated with reactant diffusion and/or surface saturation effects that slows down the reaction kinetics. The irreversibility of the electrochemical aryl iodonium reduction is visible in the CV by the absence of an oxidation peak during the reverse scan. In the second and third cycles, the recorded cathodic current is lower than in the first cycle, suggesting a surface saturation effect resulting from the near‐complete functionalization that occurs during the first cycle.

After removing the electrolyte solution, the graphene surface was characterized by scanning Raman microscopy (SRM). In comparison to pristine graphene that exhibits an intense *G* band at 1582 cm^−1^ and a prominent *2D* band at about 2680 cm^−1^, functionalized graphene shows an additional *D* band at 1350 cm^−1^ being indicative for *sp^3^* defect sites that were introduced to the *sp^2^* lattice upon binding the functional addends. Due to the dependency of the *D* band intensity *I_D_* on the defect density [30, 31], the *I_D_/I_G_* ratio is a suitable parameter to picture variations in the degree of functionalization. The evenly distributed high *I_D_/I_G_* ratio measured after the electrochemical treatment (shown in Figure 2c) confirms the large‐scale homogeneous functionalization of the entire graphene area that was exposed to the reactant solution.

While the electrochemical approach produces large areas of functionalized surfaces, the photochemical approach allows a more precise lateral control of the modified area. This is achieved by moving a focused laser across the graphene surface in specific patterns, which locally triggers the functionalization reaction and allows dynamic control over the size and shape of the illuminated areas. By adjusting the parameters of the laser, such as the excitation wavelength *λ*, the laser power *P_L,_* and the local irradiation time *t*, the degree of functionalization introduced to the graphene lattice can be tuned [17]. The photo‐electrochemical functionalization of graphene, which combines both techniques, was performed in a specially designed electrochemical cell that allows laser irradiation and “read‐out” via a confocal Raman spectrometer (see Figure S1). For this, different electrochemical potentials were applied during the step‐wise laser‐triggered “writing” process, starting from the open‐circuit potential (ocp) at 0.15 V vs. Ag|AgCl and varying stepwise in both negative and positive directions. For simplicity, the applied potential will be expressed throughout this work as the voltage difference relative to the ocp value. Based on the CV control experiment (Figure S5), a stable electrochemical window from −0.4 to +0.3 V vs. ocp was identified in which no currents correlated to surface functionalization were observed. Accordingly, the potential window used for the combined laser‐triggered functional group “writing” experiments was set within this range to ensure that no unintended functionalization occurs from an electrochemical reduction alone.

**FIGURE 3:**
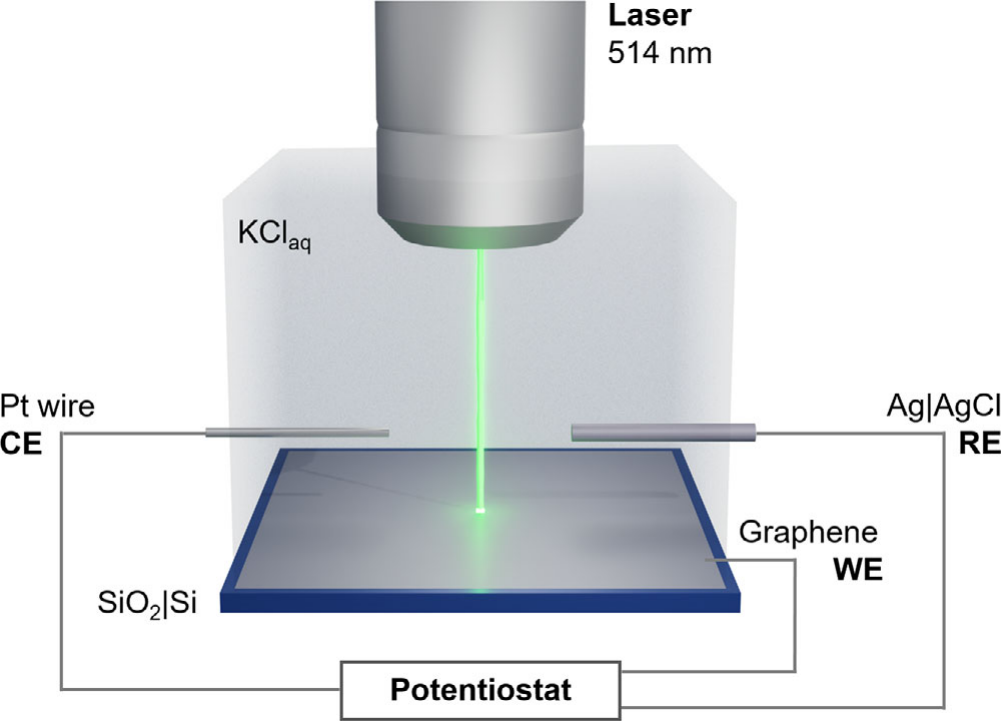
Experimental setup for combined electrochemical and laser‐triggered functionalization (WE – working electrode, CE – counter electrode, RE – reference electrode).

The electrochemical cell shown in Figure 3 was mounted in a Raman spectrometer equipped with a 40x immersion objective, enabling optimized laser focusing and enhanced lateral resolution. Three different sets of laser parameters with varying laser powers (*P_L_ * =  0.121, 0.482, and 1.121 mW) at an excitation wavelength of *λ * =  514 nm and a constant local irradiation time of *t*  =  1 s were chosen to functionalize three distinct domains of graphene to a low, medium, or high degree at ocp. To investigate the influence of the electrochemical potential on the laser “writing” process, laser “writings” were generated with these three sets of laser parameters at each potential step (*ΔV*  =  0.1 V) next to the initial pattern. After removing the reactant solution and rinsing the sample with water to prevent any unwanted subsequent laser‐triggered functionalization, the functionalized areas were characterized in detail by SRM.

**FIGURE 4:**
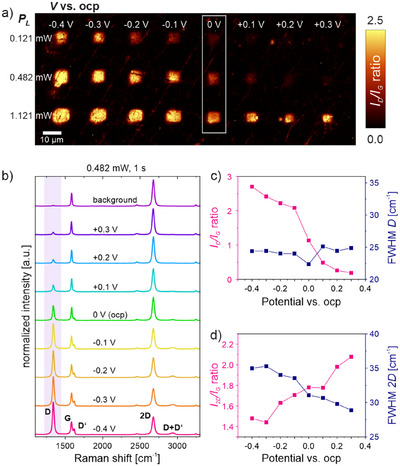
(a) *I_D_/I_G_* “readout” Raman map illustrating the spatially controlled laser‐triggered “writing” of graphene with *bis*(4‐fluorophenyl)iodonium triflate under alternating applied electrochemical potentials (“writing” parameter: *λ*  =  514 nm, *P_L_ * =  0.121, 0.482, and 1.121 mW, *t*  =  1 s). (b) Representative normalized mean Raman spectra of the functionalized areas with 0.482 mW laser power, along with the corresponding evolutions of the intensity ratios and full width half maximum (FWHM) values of the *D *band (c) and *2D* band (d). (see Figures S6–S8 for further details).

Figure 4a shows the *I_D_/I_G_* Raman “readout” mapping of the “laser‐functionalized” areas under different applied potentials and laser powers. At the starting potential of 0 V vs. ocp (highlighted by the white box), the color gradient reflects the three levels of functionalization (increasing from top to bottom), with higher degrees of functionalization indicated by brighter colors. These three domains functionalized at 0 V vs. ocp can be referred to as an internal reference system that allows to directly investigate changes in the reaction efficiency of the laser‐induced functionalization upon altering the externally applied electrochemical potential. When the applied electrochemical potential was adjusted stepwise to more negative values, the *I_D_/I_G_* ratio clearly increased, represented by brighter regions in the mapping. In contrast, the presence of more positive potentials yielded darker regions, indicating a lower degree of functionalization at the respective laser power and irradiation time.

Exemplary normalized mean Raman spectra of the areas irradiated with a laser power of 0.482 mW show a progressive increase in *D* band intensity from positive to negative potentials, as presented in Figure 4b. This increase in *I_D_/I_G_* ratio (Figure 4c) is accompanied by a decrease in *2D* band intensity and a slight increase in the full width at half maximum (FWHM) values (Figure 4d). Kelvin probe force microscopy (KPFM) images reveal a decline in surface potential for the functionalized areas, indirectly proving the local attachment of fluorophenyl moieties (refer to Figure S9). Overall, the degree of functionalization increases with the simultaneous application of more negative potentials, demonstrating a direct dependence of the laser‐induced functionalization process on the electrochemically tuned electronic states of graphene. This allows for the use of more gentle laser irradiation conditions while still yielding comparable degrees of functionalization, simply by applying a more negative potential with lower laser powers. For example, an *I_D_/I_G_* ratio of ∼ 1.7 is reached for *P_L_*  =  1.121 mW at ocp, while by applying a negative potential of 0.4 V vs. ocp allows this *I_D_/I_G_* ratio to be exceeded even when using only 10% of the initial laser power (*I_D_/I_G_* ratio ∼ 2 for *P_L_*  =  0.121 mW at 0.4 V vs. ocp) (refer to Figure S8). This amplification of the functionalization rate at more negative potentials is of especially high interest for the area‐selective functionalization of sensitive samples that could undergo degradation with higher laser powers.

To interpret the observed correlation between the applied electrochemical potential and the resulting degree of functionalization, the underlying reaction principles must be considered. According to the proposed mechanism of the laser‐triggered “writing” process, the precursor molecule is not directly excited by the incident laser light. Instead, graphene acts as a photosensitizer. In this context, laser irradiation generates excited “hot” electrons in graphene that transiently populate high‐energy states in the conduction band, at energy levels beyond the excitation energy of the laser. This is possible since their distribution corresponds to a much higher electron temperature than the lattice temperature [16, 17, 32]. A fraction of these high‐energy electrons is subsequently transferred to adsorbed precursor molecules, initiating the mandatory radical formation, which represents the rate‐determining step in the overall reaction kinetics of the covalent functionalization process [33].

**FIGURE 5:**
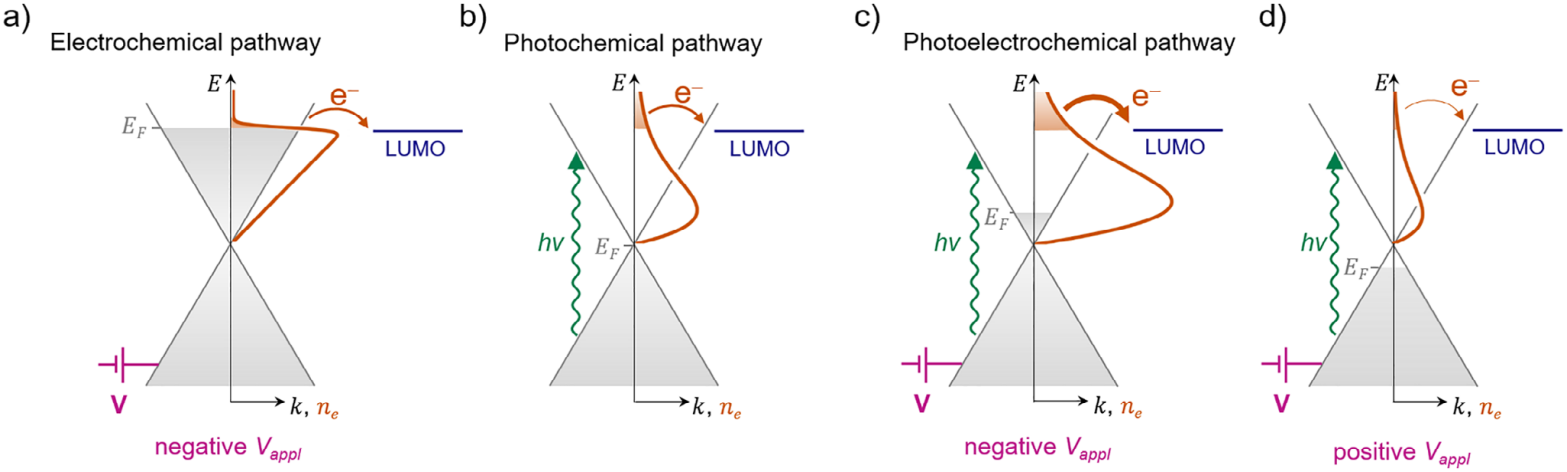
Proposed mechanism of (a) the electrochemical heterogeneous electron transfer, where the Fermi level *E_F_* in graphene is shifted by electrochemical potential *V_appl_*, (b) the photochemical excitation via laser irradiation *hν*, and (c) the photoelectrochemically activated electron transfer from graphene electrons into the lowest unoccupied molecular orbital (LUMO) of the reactant molecule for negative applied potentials and (d) for positive applied potentials. The electron distribution of graphene *n_e_* (orange line) changes to a “hot” electron profile upon photochemical excitation in (b), (c), and (d), which increases the fraction of high‐energy electrons above the LUMO that can undergo the electron transfer. The simultaneously applied potential *V_appl_* in (c) modifies the “hot” electron *n_e_* curve by increasing the Fermi level *E_F_* (left) via a negative *V_appl_* or decreasing it (d) via a positive *V_appl_* (see Section S2 for further details). The cones sketch the electron band structure near the K point of the graphene Brillouin zone; grey filling indicates occupation of the states by electrons, according to the position of the Fermi level.

Applying a negative electrochemical potential to the graphene increases the Fermi level, which corresponds to n‐doping [34]. Conversely, positive electrochemical potentials lower the Fermi level, which correlates to p‐doping of graphene. For the case of a purely electrochemical process, the Fermi level needs to be shifted to an energy level that is above the LUMO of the precursor molecule, in order to facilitate transitions of the electrons located in the graphene into the LUMO (Figure 5a). If the Fermi level cannot be shifted to values above the LUMO, such a transfer is not possible, and electrons have to be excited into higher energy level, e.g., via laser irradiation. Electrons that occupy states in the upper part of the Dirac cone as a result of the Fermi‐level shift cannot be further excited, as no electronic states at higher energies are available for light‐induced vertical transitions within the graphene band structure. Consequently, these electrons cannot be transferred into the LUMO of the precursor molecule. Density‐functional theory (DFT) computations of the electronic states of the precursor molecule show that the LUMO is well outside the range of energies that can be reached by direct excitation with the laser used here, see Section S2.1 for details. However, as shown previously, laser irradiation induces the formation of excited “hot” electrons in graphene (Figure 5b) [17, 35, 36, 37]. Owing to their broad thermal energy distribution (orange line in Figure 5b), a fraction of these electrons possesses sufficiently high energies to undergo an electron transfer into the LUMO of the reactant, thereby leading to a reduction of the precursor molecules. By increasing the Fermi level, the fraction of “hot” electrons excited into energy levels higher than the LUMO level is likewise increased, while a decrease in Fermi energy is related with a decrease in the fraction of “hot” electrons that are higher in energy than the LUMO of the reagent molecule. More negative potentials (i.e. higher Fermi levels) in turn facilitate the electron‐transfer rate/kinetics and results in a higher degree of functionalization (Figure 5c). Conversely, at positive applied potentials, the fraction of high‐energy electrons is reduced, although statistically not zero (Figure 5d). In this potential regime, heterogeneous electron transfer remains thermodynamically possible; however, surface functionalization is experimentally observed only at higher laser powers, which compensate for the reduced electron population by increasing the lifetime of the “hot” electrons [37]. At low laser powers and positive potentials, the heterogeneous electron‐transfer rate becomes too slow, and significant graphene functionalization is no longer observed. These considerations provide a qualitative rationale for the experimentally observed potential‐dependent modulation of the laser‐induced functionalization process.

These results clearly demonstrate the strong potential of our photoelectrochemical functionalization strategy. The approach not only enables precise lateral control over the covalent functional group binding and allows amplification of the degree of functionalization when applying the same set of laser “writing” parameters through the application of negative potentials (see Figure 4), but also provides a direct route toward in situ “off”‐switching of the laser‐triggered reaction by applying positive electrochemical potentials.

**FIGURE 6:**
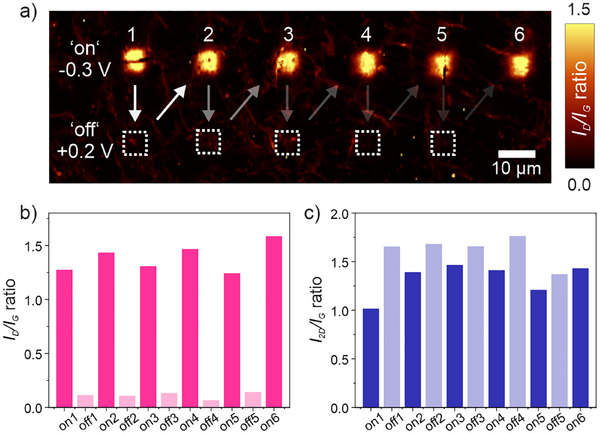
(a) *I_D_/I_G_* “readout” Raman map of the performed laser “writing” under “on” (−0.3 V vs. ocp) and “off” (+0.2 V vs. ocp) conditions. (b) Evolution of the *I_D_/I_G_* ratio during consecutive switching between “on” and “off” conditions, showing consistent high *I_D_*/*I_G_* values for all “on” steps and values close to zero for “off” steps. (c) Corresponding *I_2D_/I_G_* ratio evolution (see Figures S10 and S11 for further details).

In order to investigate this “off” switching behavior, a defined laser “writing” parameter set was selected, using −0.3 V vs. ocp as the “on” and +0.2 V vs. ocp as the “off” potential. A multi‐step sequence was then conducted, in which the potential was alternated between “on” and “off” states. At each step, laser‐triggered functional group “writing” was performed to probe the robustness and reproducibility of the switching performance. Figure 6 presents the resulting *I_D_/I_G_* and *I_2D_/I_G_* ratios of the laser‐irradiated regions of each “writing” step, revealing an *I_D_/I_G_* ratio of approximately 1.3 for all “on” areas as well as an *I_D_/I_G_* ratio close to zero with applied “off” potential. Moreover, the *I_2D_/I_G_* ratio exhibits a corresponding decrease in the functionalized regions, further confirming the successful covalent modification under “on” conditions. In addition to demonstrating the reversible deactivation of the photoelectrochemical functionalization reaction in multiple steps, this sample likewise allows to gain first insights into the reproducibility of the functional group grafting under identical parameters and potential local variations. The mean *I_D_/I_G_* ratios for the functionalization under “on” conditions presented in Figure 6b varies between 1.3 and 1.6, visualizing the limits of accuracy. To illustrate the distribution of *I_D_/I_G_* ratios within the individual domains, histograms are shown in Figure S12, revealing a relatively broad distribution of *I_D_/I_G_* ratios with the majority of data points between 0.5 and 2 for all six domains. Compared to solid‐state laser “writing” [38], this reveals a lower homogeneity within the functionalized areas, which is presumably related to pronounced laser scattering effects due to the liquid phase approach. We have previously reported a resolution limit of ∼2 to 3 µm for a similar functionalisation approach and expect this resolution to be adoptable for the presented photoelectrochemical functionalization [29].

**FIGURE 7:**
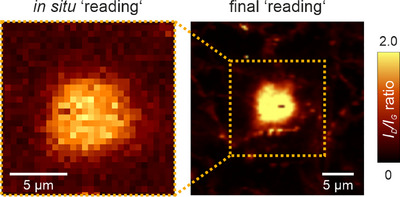
In situ *I_D_/I_G_* Raman “readout” map recorded under “off” conditions (+0.2 V) (left), and larger ex situ Raman map of the same region to assess whether the initial in situ “readout” performed under “off” conditions affected the surrounding non‐irradiated background (see Figure S13 for further details).

The reproducible “on” and “off” switching achieved by adjusting the electrochemical potential highlights the practical utility of this external control strategy. Building on the reliable and effective suppression of the laser‐induced covalent graphene functionalization in the “off” state, we addressed one of the most prominent challenges of the laser‐triggered “writing” procedure. As outlined above, functionalized graphene is typically characterized by Raman spectroscopy, which itself involves laser irradiation—in fact, both the “writing” and “reading” steps can be performed using the same instrument. However, Raman “readout” that is performed in the continued presence of the reactant can lead to unintended background functionalization. So far, this can only be prevented by the removal or irreversible deactivation of the reagent and would require a new supply of reagent to continue the functionalization process after characterization. Here, the ability of electrochemically switching the reactivity into a reversible deactivated “off” state provides a straightforward solution to analyze the induced functionalization using in situ Raman spectroscopy by suppressing covalent functionalization during Raman analysis and reactivating the reactivity for a further precise functionalization. Figure 7 shows the Raman *I_D_/I_G_* map of an in situ “readout” performed with the reactant solution still present and an applied potential of +0.2 V. A previously functionalized region is clearly visible as a brighter contrast, corresponding to a higher *I_D_/I_G_* ratio relative to the unmodified background. After removing the reactant solution and thoroughly rinsing the sample with water and isopropanol, a second ex situ “readout” was performed over a larger area to determine whether the in situ measurement had caused any changes to the surrounding regions. Both the Raman map in Figure 7 and the normalized mean Raman spectra in Figure S13 show no difference between the area irradiated during the in situ characterization (graphene at positive potential of +0.2 V) and the adjacent comparison region. This confirms that in situ characterization can be carried out without inducing unintended modifications of the graphene lattice. Furthermore, it enables a stepwise “writing” process in which each step can be monitored by in situ Raman measurements. This unprecedented capability significantly enhances the relevance and practical applicability of the presented photoelectrochemical functionalization approach of graphene.

## 3 Conclusion

In summary, we have developed a photoelectrochemical strategy that combines spatially resolved laser‐triggered graphene functionalization with electrochemical control over the reaction driving force. The laser “writing” approach offers lateral precision and flexible pathways for covalent addend binding, where the electrochemical gating allows for a systematic tuning of the electron‐transfer kinetics through the variation of the applied potential. Within an electrochemical window that excludes an electrochemically triggered grafting, the degree of functionalization can be varied in a controlled manner by altering the applied potential during the laser‐triggered precursor activation. More negative potentials relative to the open‐circuit potential enhance an addend binding, whereas more positive potentials reduce the grafting density. This potential‐dependent modulation enables a reproducible switching between defined “on” and “off” states of the laser‐triggered reaction. Notably, the suppression of the addend binding under positive bias enables, to our knowledge, the first reversible electrochemical deactivation of laser‐induced graphene functionalization. This directly allows for an in situ Raman characterization in the continued presence of the precursor solution. Therefore, the need for an intermediate removal of the reactant solution and its subsequent reapplication can be eliminated, thereby enabling an uninterrupted, stepwise monitoring of the functionalization process directly during or after laser “writing”. By combining the spatial selectivity with a voltage‐controlled reaction tuning, this approach establishes a dual‐control framework and introduces electronically gated covalent chemistry as a design principle for 2D materials. We expect our approach to be transferable to other precursor classes with a similar reaction mechanism, such as widely used diazonium compounds. In particular, the ability to slow down the reaction kinetics of such highly reactive precursors by applying a positive potential to the graphene, thereby increasing the energy barrier between the reagents, could offer both kinetic and lateral control over an otherwise spontaneous reaction and open the pathway to a broader range of possible graphene addend functionalities [34, 36, 37, 39, 40, 41, 42, 43, 44].

## Conflicts of Interest

The authors declare no conflicts of interest.

## Supporting information




**Supporting File**: advs75454‐sup‐0001‐SuppMat.pdf.

## Data Availability

The data that support the findings of this study are openly available in zenodo at http://doi.org/10.5281/zenodo.19664117.
