# Supplementary material for: Electrochemical Modulation of Laser‐Induced Covalent Functionalization of Graphene
Source: Adv Sci (Weinh). 2026 Apr 27;13(39):e75454. doi: 10.1002/advs.75454 (PMC13335470; doi:10.1002/advs.75454)
Supplement: Supplementary file 1 — Supporting File: advs75454‐sup‐0001‐SuppMat.pdf. [file ADVS-13-e75454-s001.pdf]

Supporting Information

Electrochemical Modulation of Laser-Induced Covalent Functionalization of Graphene

Tamara Nagel,+[a] Linda Feuerstein,+[b] Kevin Gerein,[a] Stefan Wolff,[c] Janina Maultzsch,[c] Andreas Hirsch*[a], Inez M. Weidinger*[b] and Frank Hauke*[a]

[a] Department of Chemistry and Pharmacy & Center of Advanced Materials and Processes (ZMP), Friedrich-Alexander Universität Erlangen-Nürnberg, Nikolaus-Fiebiger-Str. 10, 91058 Erlangen, Germany

[b] Chair of Electrochemistry, Technische Universität Dresden, Zellescher Weg 19, 01069 Dresden, Germany

[c] Department of Physics, Chair of Experimental Physics, Friedrich-Alexander-Universität Erlangen-Nürnberg, Staudtstr. 7, 91058 Erlangen, Germany

1. Experimental Section

1.1. Materials and Chemicals

CVD graphene was purchased from ACS Materials Inc. as trivial transfer graphene with PMMA coating. Si/SiO_2 wafers (300 nm thick silicon oxide layer) were obtained from Fraunhofer e.V. (Germany) and cut to 0.5 x 0.5 cm substrates. Bis-(4-fluorophenyl) iodonium triflate was bought from Merck Sigma-Aldrich Chemie GmbH. All other chemicals and solvents were purchased from Merck Sigma-Aldrich Chemie GmbH, Carl Roth GmbH, and VWR International GmbH and, if not stated otherwise, used with no further purification.

1.2. Sample Preparation

Transfer of CVD Graphene on Si/SiO_2 Substrate: The target substrate was cleaned beforehand by iterative spin coating with acetone (99 %, HPLC grade) and isopropanol (99 %, HPLC grade). The CVD graphene/ PMMA film located on the carrier material was moistened (H_2O) carefully to loosen the graphene-carrier bond. Thereafter, it was released into double distilled water, letting the graphene float on the surface. To divide the graphene into smaller pieces the floating sheet was picked up using a filter paper, cut with sharp scissors, and immediately re-floated on the double distilled water surface. Afterwards, the pre-cleaned substrate (Si/SiO_2 wafer) was placed in the water underneath the cut floating film and cautiously pulled out of the water to position the film on the surface. Then, the sample was dried and heated to 130 °C for one hour to remove any residual water trapped under the

graphene lattice. The protective PMMA layer was then gently removed using acetone vapor at 60 °C for 2 hours.

1.3. Electrochemical Experiments

The setup of the self-designed electrochemical cell suitable for laser 'writing' is shown in Figure S1. Graphene was connected as the working electrode (WE) via a copper adapter that was insulated from the electrolyte solution using an O-ring with a diameter of 2.5 mm. The area of graphene exposed to the electrolyte solution is 4.91 mm^2. A Pt wire was used as counter electrode (CE) and an Ag|AgCl|KCl (3 M) electrode served as reference electrode (RE). The aqueous electrolyte solution was prepared with 0.1 M KCl as support electrolyte, giving an open circuit potential (ocp) value of 0.15 V vs. Ag|AgCl|KCl (3 M). Laser 'writing' under applied potential was performed using chronoamperometric measurements at a Vertex.One potentiostat from Ivium Technologies, with applied potentials ranging from –0.25 V to +0.45 V vs. Ag|AgCl. Cyclic voltammetry measurements were performed on a BioLogic SP-300 potentiostat.

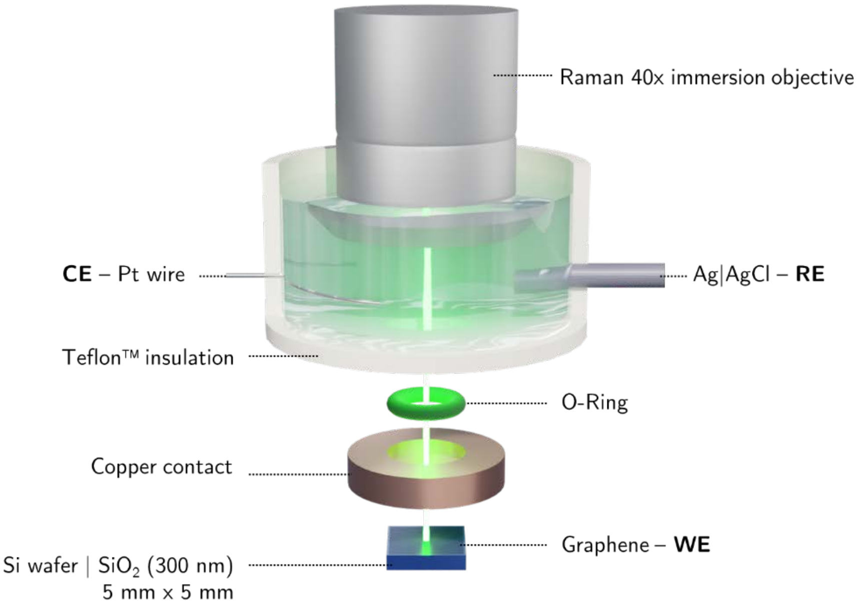

Figure S1: *In-situ* electrochemical cell for combined electrochemical and laser 'writing' functionalization of graphene (RE – reference electrode, CE – counter electrode, WE – working electrode).

1.4. Raman Spectroscopy – *in-situ* laser 'writing'

The laser 'writing' procedures as well as Raman spectroscopy for *in-situ* characterization were performed on a S&I Monovista CRS+ confocal Raman spectrometer equipped with a motorized XYZ table (Scan IM 200x200, Märzhäuser GmbH & Co. KG, Germany) and a liquid nitrogen cooled CCD detector. The laser was focused on the graphene surface using a 40x Water immersion objective "W Plan-APOCHROMAT" (Zeiss) DIC VIS-IR with numeric aperture NA = 1.0. All experiments were carried out with a laser excitation wavelength of λ = 514 nm, using a Cobolt Fandago laser (Hübner Photonics). Spectra were obtained using a holographic grating of 300 grooves/mm.

1.5. Raman spectroscopy - characterization

The Raman spectroscopy for the characterization was performed on a WITec alpha300R confocal microscope equipped with a motorized XYZ table (Δxy_{min} = 10 nm, Δz_{min} = 30 nm) and a high-sensitivity CCD detector (1,024 x 127, 80 spectra per second). The Raman system was coupled to an optical microscope operating both in bright and dark field, as well as an internal calibration source. The setup was controlled by the WITec Control software, equipped with EasyLink, facilitating control over spatial coordinates, and TruePower function, enabling a distinct adjustment of the laser power (P_L) on the sample surface with an accuracy of <0.1 mW, ensured by laser power measurements in the optical fiber. This has also been confirmed by an external P_L calibration. Measurements were conducted with 100x Zeiss EC "Epiplan-Neofluar" DIC (NA = 0.90) and LD 50x Zeiss EC "Epiplan-Neofluar" DIC (NA = 0.5) objectives and grating of 600 grooves/mm. The acquired data were evaluated with the WITec Project software. All measurements were carried out with a laser excitation wavelength of λ = 532 nm (DPSS laser with a maximum PL of 30 mW), that was coupled to a custom long-wavelength filter prior to coupling to the Raman optics. The focused laser spot size is 300 nm according to Witec.

1.6. Scanning Probe Microscopy

Scanning probe microscopy was carried out using a Bruker Dimension Icon 3 microscope in AM-KPFM mode (amplitude modulated kelvin probe force microscopy). Bruker SCM-PIT-V2 probes (platinum-iridium coating) on antimony doped Si cantilevers were used to obtain the KPFM images with 512 x 512 pixels or 1024 x 1024 pixels resolution (scan rate: 0.2 Hz). The operative software was NanoScope 9.4 and the received data were evaluated with Gwyddion.

1.7. Experimental Procedure

General Write/Read Functionalization Procedure: In a first step, pristine graphene on a Si/SiO_2 substrate was pre-characterized by Raman spectroscopy to ensure the appropriate quality of the graphene layer. After mounting the sample in the beforehand described electrochemical cell and adding the electrolyte solution (air bubbles have to be avoided to ensure a good optical resolution) a suitable area of the graphene layer on Si/SiO_2 has been selected by optical microscopy. The selection criteria were mainly based on an optically mostly defect free surface combined with some specific sample features such as holes, wrinkles, multilayer islands or selective surface contamination as natural markers. To ensure that the functionalized areas can be precisely localized again in the further course of the investigations optical images of the selected area with different magnifications were taken. This is a very important step, as the covalent functionalization of graphene introduced later does not change the optical appearance of graphene, making it impossible to find the laser functionalized area without detailed process planning and recording.

To ensure a sufficient reactivity of the reactant solution, several Raman test measurements were performed in the surrounding area, which already need to show functionalization of graphene due to an increasing *D* band. For the photoelectrical functionalization process, a specific pattern was designed in advance, including the laser writing parameters, the dimensions and positions of the areas and the planed electrochemical potential. The coordinate system of the Raman setup allows for a precise positioning of the areas. The laser-triggered covalent graphene functionalization with altering electrochemical potential was performed in a point raster mode. In general, the following parameters can be adapted: laser excitation wavelength (λ), laser power (P_L), irradiation time of each point (t), the dimensions in x and y, the distance between points, the position according to the coordinate system and the applied potential. After the actual photoelectrical functionalization procedure, the electrochemical cell was removed from the Raman stage. The graphene sample was demounted from the cell and rinsed with isopropanol and water to remove residual reactant on the surface that could cause unwanted modification of the graphene lattice during the readout process. For the Raman readout characterization, the sample was reinstalled in the Raman setup and the patterned area was identified using the previously obtained optical images. To confirm the completeness of the washing procedure, several single point Raman measurements were performed using similar parameters as for the reactivity study of the reactant solution, which must present typical pristine graphene Raman spectra. The Raman readout measurement was then performed in an area selected to be several µm larger than the functionalized areas. Standard parameters for the Raman readout are: $\lambda = 532$ nm, $P_L = 5$ mW, $t = 0.5$ s or 1 s as acquisition time per point and 0.33 µm as point spacing.

1.8. Statistical Analyis

The data analysis of ‘in-situ’ mapping was carried out using Python code executed in Jupyter Notebooks. All other characterization (= ‘readout’) Raman data was generated and processed using Witec Control Six. Mappings, mean spectra and histograms were exported and further processed with Origin 2021. Spectral preprocessing includes spike-removal, baseline-correction and normalization of the mean spectra to the *G* band. Intensity ratios were calculated from the absolute peak heights of the mean spectra. Histogram evaluation was done by Gauss fitting.

2. Mechanistic Considerations

We performed density-functional theory (DFT) calculations of the precursor molecule Bis(4-fluorophenyl)iodonium triflate and graphene in order to obtain the alignment of the LUMO and HOMO of the precursor molecule with the electron bands of graphene. We further discuss the Fermi-level shift by the electric potential and the influence of the Fermi-level shift and laser excitation on reaction kinetics. As we will show below, our quantitative evaluation gives an upper limit for the energy difference between the molecule's LUMO and the Fermi level of undoped graphene.

2.1. DFT calculations

All density functional theory (DFT) calculations were performed using the Quantum ESPRESSO suite[1,2] and utilizing norm conserving pseudopotentials from the SPMS library[3] and the Perdew-Burke-Ernzerhof (PBE) exchange–correlation functional[4] which is known to underestimate the electronic band gap. Structural optimizations were performed until the forces exerted on each atom were reduced to below 0.005 eV $Å^{-1}$. The lattice vector of the graphene unit cell perpendicular to the graphene plane was chosen to be 35 Å in order to avoid interactions between neighboring cells. The calculations were performed with well converged parameters. The Brillouin zone was sampled by a 14 × 14 × 1 Monkhorst-Pack *k*-point grid and a plane wave cutoff of 80 Ry (1088.46 eV) was used.

Bis(4-fluorophenyl)iodonium triflate was placed inside a cube of side length 35 Å for which the wave functions could be chosen as real, and thus no *k*-point sampling was used. Bis(4-fluorophenyl)iodonium triflate is a molecule which has a positive charge located at the iodine atom, which was included in the relaxation calculation. Because of the way Quantum ESPRESSO treats nonneutral unit cells, a more accurate determination of the electronic states relative to the vacuum level is achieved by using a charge-neutral unit cell. For this reason, the band gap between the highest occupied molecular orbital (HOMO) and the lowest unoccupied molecular orbital (LUMO) can only be used as an upper limit, whereas by including the positive charge of the molecule, the HOMO-LUMO-gap is reduced by about 0.5 eV. The positions of the HOMO and LUMO of Bis(4-fluorophenyl)iodonium triflate were positioned within the electronic band structure of graphene by aligning the vacuum potential of graphene and the molecule. To obtain the respective vacuum levels, the sum of the potential caused by the nuclei

and the Hartree potential, was calculated and averaged along the direction of the vacuum within the unit cell.

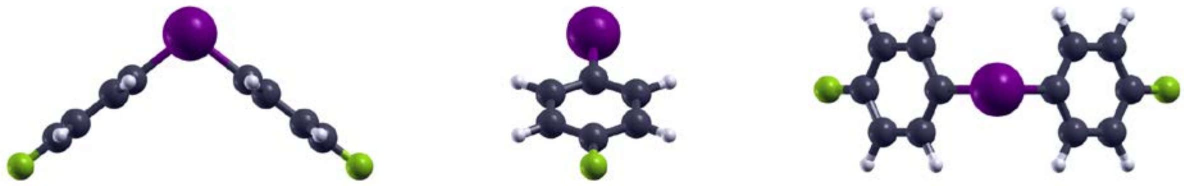

Figure S2: Optimized structure of Bis(4-fluorophenyl)iodonium triflate from three different viewing angles. Carbon atoms are shown in black, fluorine in lime green, iodine in purple, and hydrogen in white.

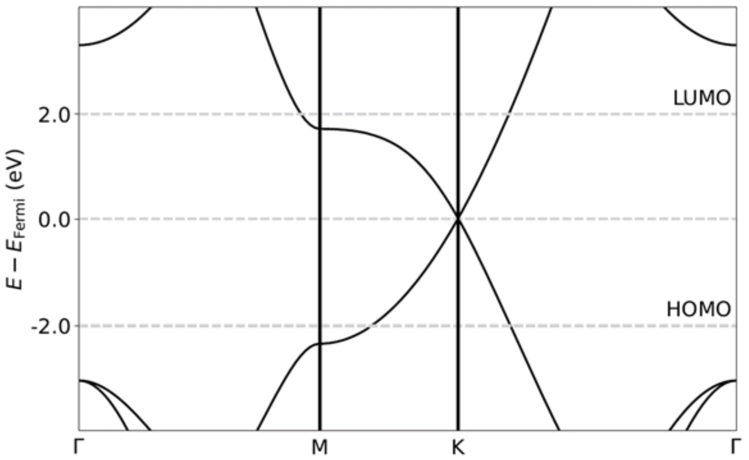

Figure S3: Calculated electronic band structure of graphene with HOMO and LUMO level of Bis(4-fluorophenyl)iodonium triflate, for a charge-neutral unit cell. HOMO and LUMO as well as graphene's Fermi energy illustrated by dashed gray lines.

2.2. Influence of the applied electric potential on the Fermi level of graphene

Appyling an electric potential to graphene changes its electronic properties via a shift of the Fermi level. A direct conversion, however, between the electrode potential that is applied against a reference electrode (given in V vs. reference potential) and the shift in Fermi energy (given in eV vs. Dirac point), is less straightforward, due to the quantum capacitor properties of graphene.[5,6] However, for the following mechanistic considerations, the Fermi-level shift under an external electrochemical potential is approximated linearly via the elementary charge *e,* serving as an upper-limit estimate. Accordingly, we approximate a Fermi level shift of +0.4 eV at the negative potential limit and -0.3 eV at the positive potential limit. It should be

emphasized that the linear approximation can deviate strongly once graphene's quantum capacitance is taken into account, particularly for low double-layer capacitances. Consequently, the absolute Fermi level shift at applied electrochemical potential may be substantially smaller than the values used here. Note that the intial doping of the graphene, resulting in an intial Fermi level away from the Dirac point is also not considered here.

Additional details on this topic can be found in Ref. 7, where Abbas et al. reported a comprehensive study on the Fermi level shift due to shifting electrode potential in various aqueous electrolytes, including comparisons of several previous studies.[7]

A quantitative evaluation of the energy requirement for the electron transfer reveals a discrepancy between the experimental electrochemical functionalization and the DFT calculations. According to the CV presented in the manuscript in **Figure 2a**, a potential of -0.5 V vs. ocp already initiates the electron transfer and thus the functionalization reaction, which implies an energy difference of 0.5 eV between the LUMO of the reagent and the initial Fermi level of graphene, at the largest approximation. In contrast, an energy difference of 2 eV was determined by DFT calculations, which, due to the method used, serves as an upper limit approximation of the band gap calculation and is described in further detail in Section 2.1. A further refinement of the theoretical model that includes experimentally relevant aspects – such as doping effects from the SiO_2/Si substrate, solvation effects of the electrolyte solution and the impact of initial defect sites on the electronic structure of graphene – could provide better quantitative agreement between experiment and theory. Such refinements, however, lie beyond the scope of this work.

2.3. Influence of shifting Fermi level on the reaction kinetics

The electron occupation of energy states $F(E)$ in graphene follows the Fermi-Dirac statistics that is dependent on the temperature T as well as the Fermi level E_F:

$$F(E) = \frac{1}{\exp\left(\frac{E - E_F}{k_B T}\right) + 1} \tag{1}$$

where k_B is the Boltzmann constant. Assuming a linear distribution of the density of states (DOS) around the Dirac Point, the electron distribution $n_e(E)$ over the energy states E is obtained by:

$$n_e(E) = F(E) \times DOS(E) \quad (2)$$

As the electron DOS increases more than linearly at higher energies, the electron density will also increase, further facilitating the laser-induced functionalization. At ambient temperature, there is a sharp edge at the Fermi level energy and only a negligible proportion of electrons populate higher eletronic states. However, it was reported before that photon absorption induces 'hot' electrons, that are not in thermal equilibrium with the surrounding lattice, meaning that their population curve is much broader and a considerable proportion of electrons populate higher electronic states.[8,9] The electrons with high enough energy above the LUMO of the precursor molecule can undergo heterogeneous electron transfer.[9]

The proportion of high energy electrons can further be modified by shifting the Fermi level E_F via an external potential as was discussed earlier in Section 2.1. The shifted Fermi level changes the population of energy states according to Fermi-Dirac statistics (1), displayed in Figure S4a, and therefore the electron distribution (Figure S4b). When shifting the Fermi level by +0.4 eV (via a negative applied potential), the proportion of 'hot' electrons at high energy states rises significantly. As a result, more electrons reach the high energy levels required for the heterogeneous electron transfer to the reactant molecule. On the opposite site, less electrons reach high electronic states, when the Fermi level is lowered by -0.3 eV via a positive applied potential. Due to the broad thermal distribution of 'hot' electrons, the proportion of electrons with energy above the LUMO is non-zero that means the electron transfer is still possible from a thermodynamic point of view. As shown in the main article, the electron transfer and a functionalization of the surface is still observed at high laser powers that compensate for the intrinsically smaller proportion of high energy electrons by inducing a higher total hot electron flux.

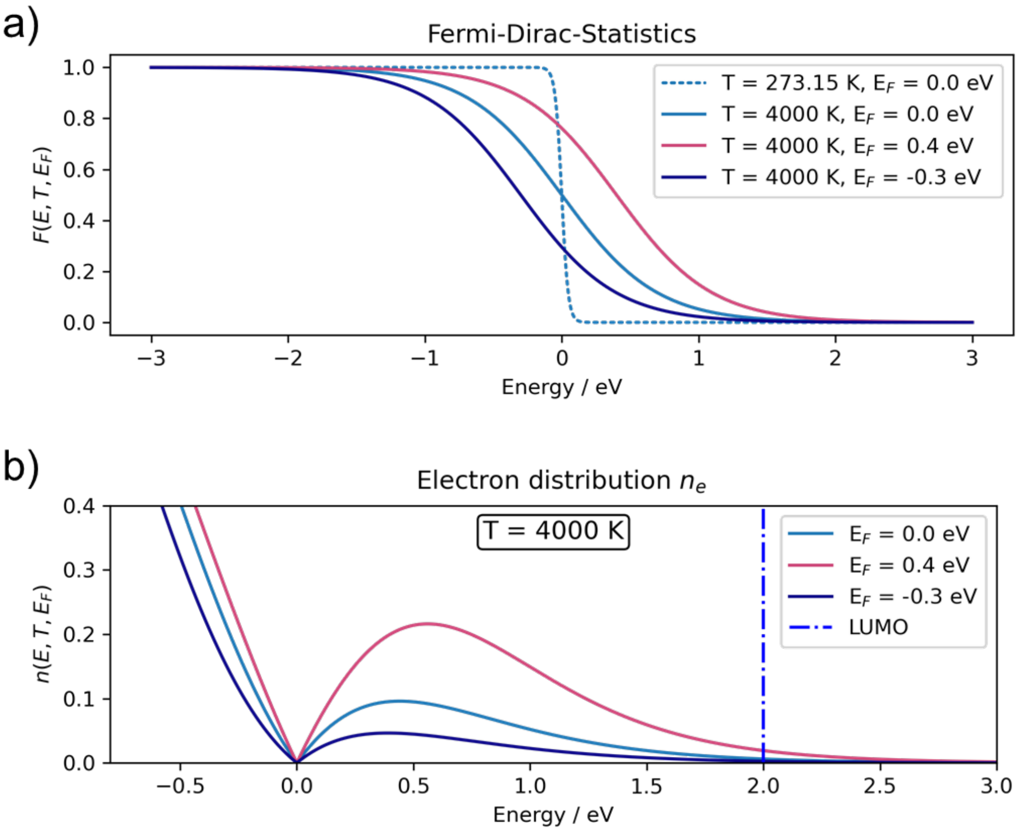

Figure S4: a) Population of energy states accroding to Fermi-Dirac statistics (equation 1) under different Fermi levels due to applying external electric potential, b) resulting electron distribution $n_e(E)$ of 'hot' electrons. LUMO energy level of bis(4-fluorophenyl) iodonium is calculated on a DFT level.

3. Data Section

3.1. Cyclic voltammetry (CV)

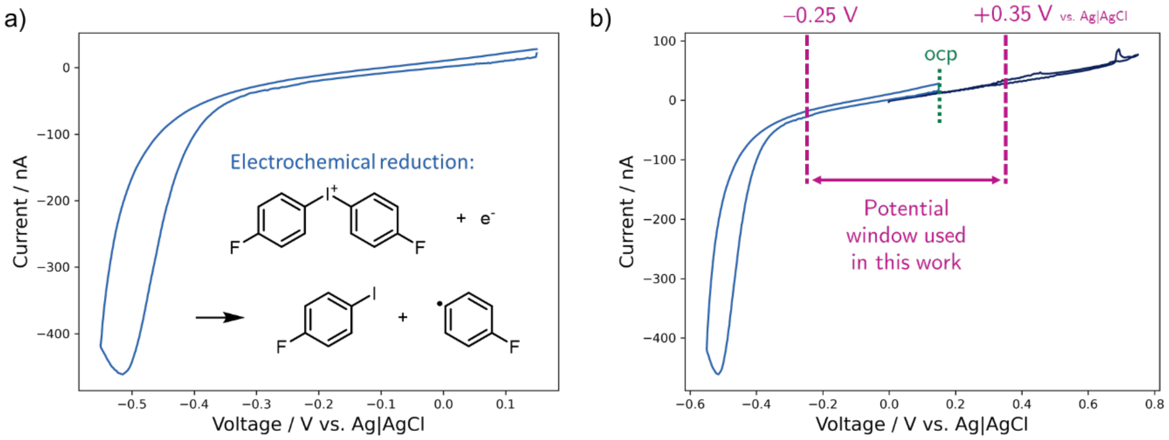

Figure S5: Cyclic voltammograms of bis(4-fluorophenyl)iodonium triflate in aqueous 0.1 M KCl electrolyte at scan rate 1 mV s^{-1}.

3.2. Parameter study – potential supported laser 'writing'

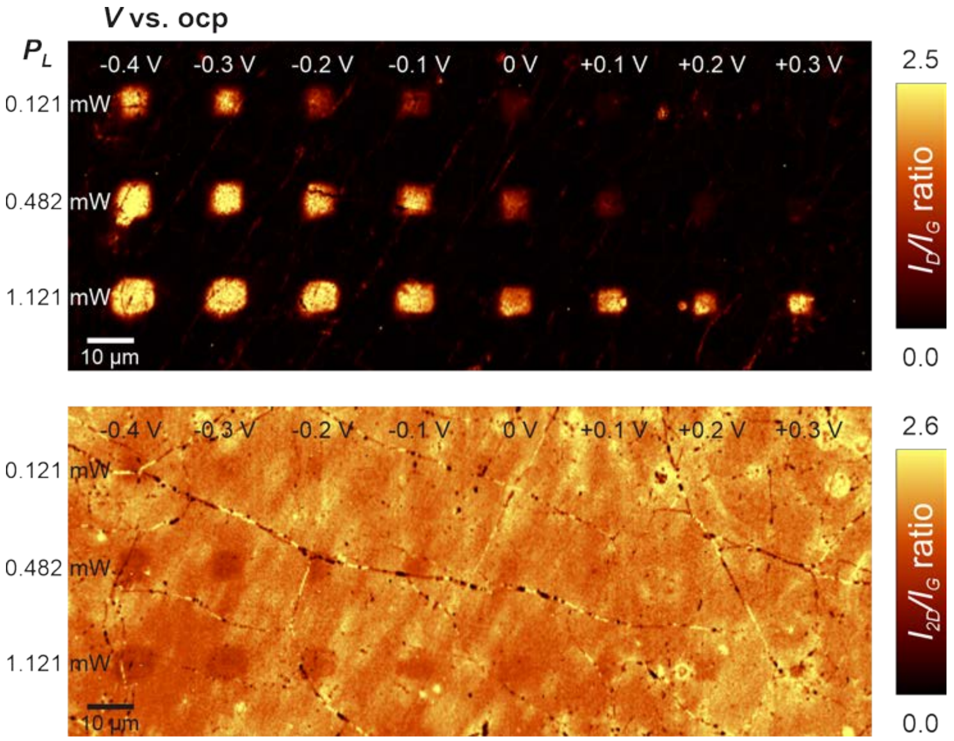

Figure S6: Raman I_D/I_G (top) and I_{2D}/I_G (bottom) mappings after the laser 'writing' performed at different electrochemical potentials.
Writing parameters: λ = 514 nm, P_L = 0.121 mW/ 0.482 mW/ 1.121 mW, t = 3 s, 5 x 5 µm, 10 x 10 points.
Reading parameters: λ = 532 nm, P_L = 5 mW, t = 0.8 s, 0.33 µm point distance.

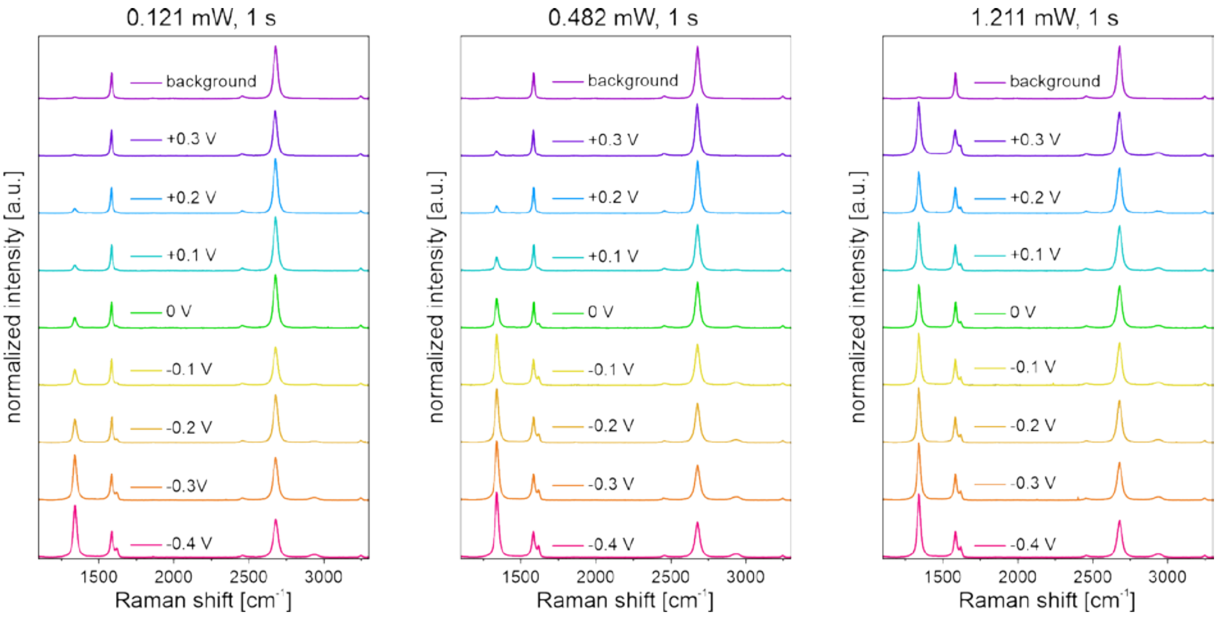

Figure S7: Normalized mean Raman spectra of all areas functionalized by laser ‘writing’ under different applied electrochemical potentials.

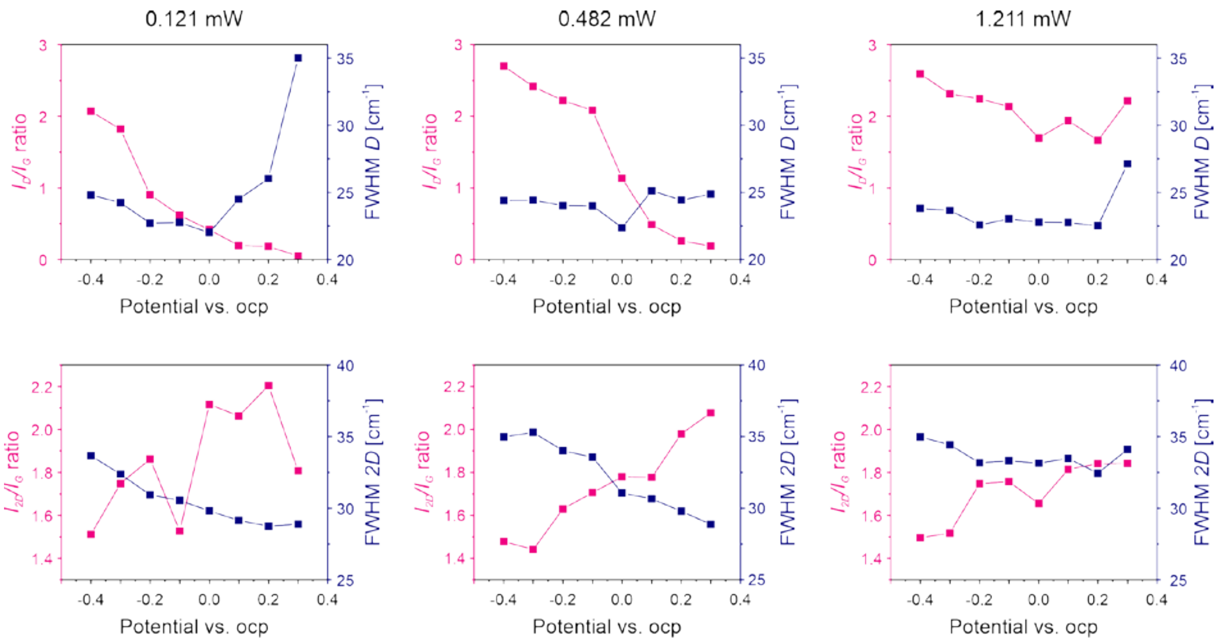

Figure S8: Evolutions of the I_D/I_G ratio and full with half maximum (FWHM) of the *D* band at varying applied electrochemical potential and used laser power (top). Corresponding trends for the I_{2D}/I_G and FWHM of the *2D* band (bottom).

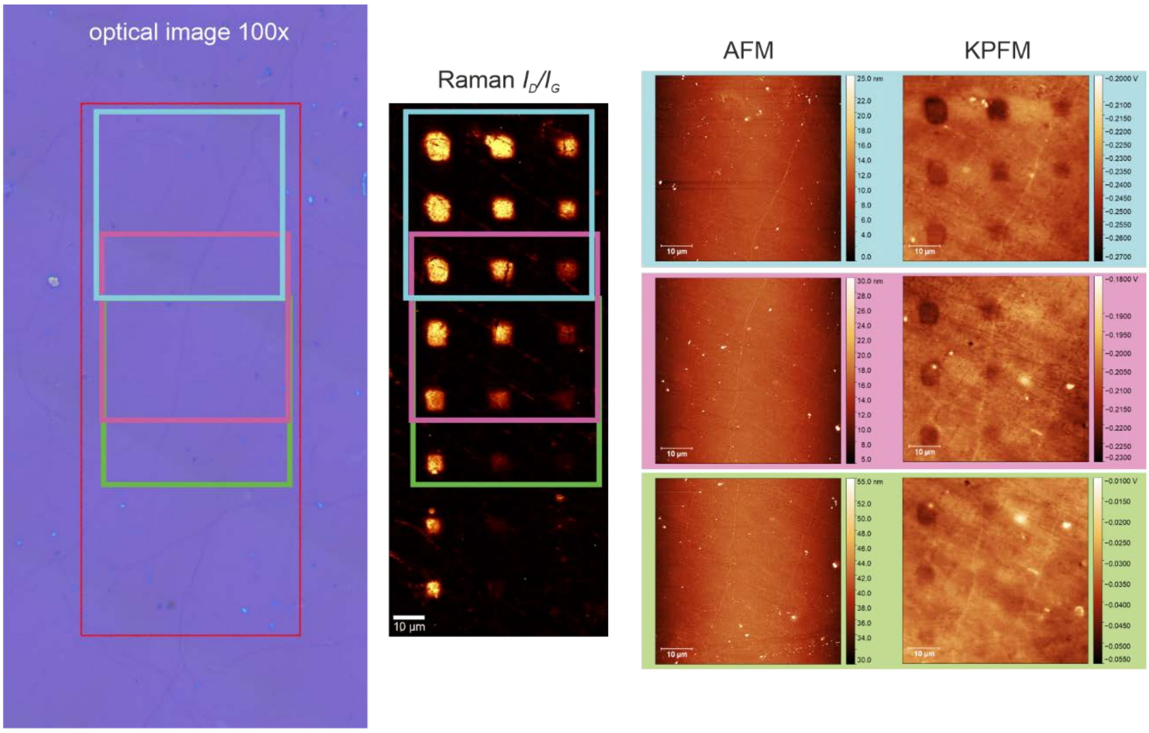

Figure S9: Optical image and Raman I_D/I_G mapping indicating the areas of the AFM and KPFM measurements visualizing the local height and surface potential of the graphene surface.

The atomic force microscopy (AFM) images reveal no significant height difference between functionalized and non-modified regions since the height increase solely caused by covalent functionalization with fluoro-phenly moieties is below the resolution limit of the system.

3.3. 'On' – 'Off' switching of laser 'writing'

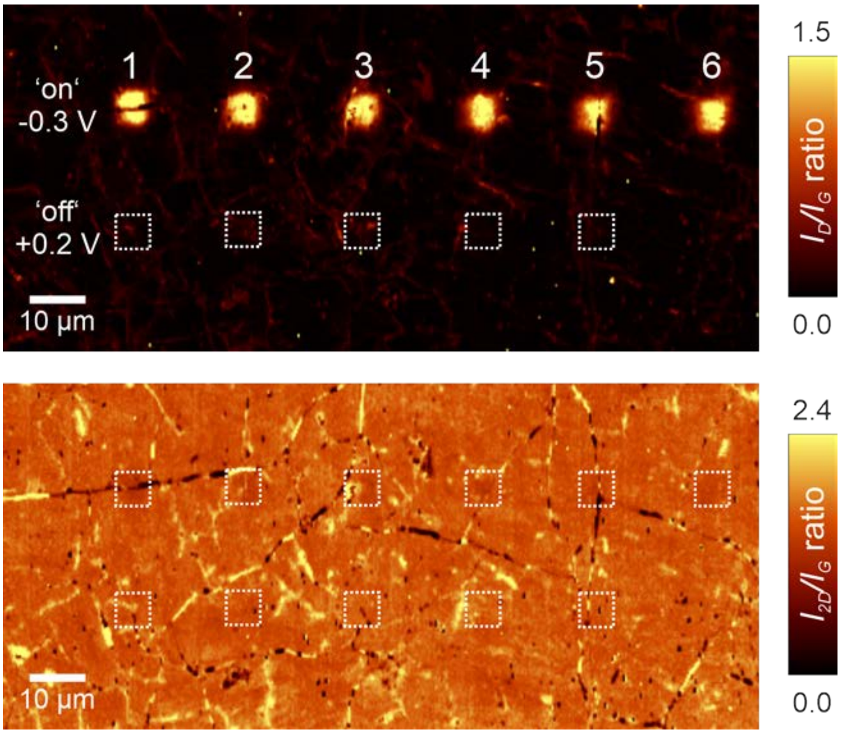

Figure S10: Raman I_D/I_G (top) and I_{2D}/I_G (bottom) mappings of the cyclic switching of laser 'writing' by controlling the electrochemical potential.
Writing parameters: λ = 514 nm, P_L = 0.482 mW, t = 1 s, 5 x 5 µm, 10 x 10 points.
Reading parameters: λ = 532 nm, P_L = 5 mW, t = 1 s, 0.33 µm point distance.

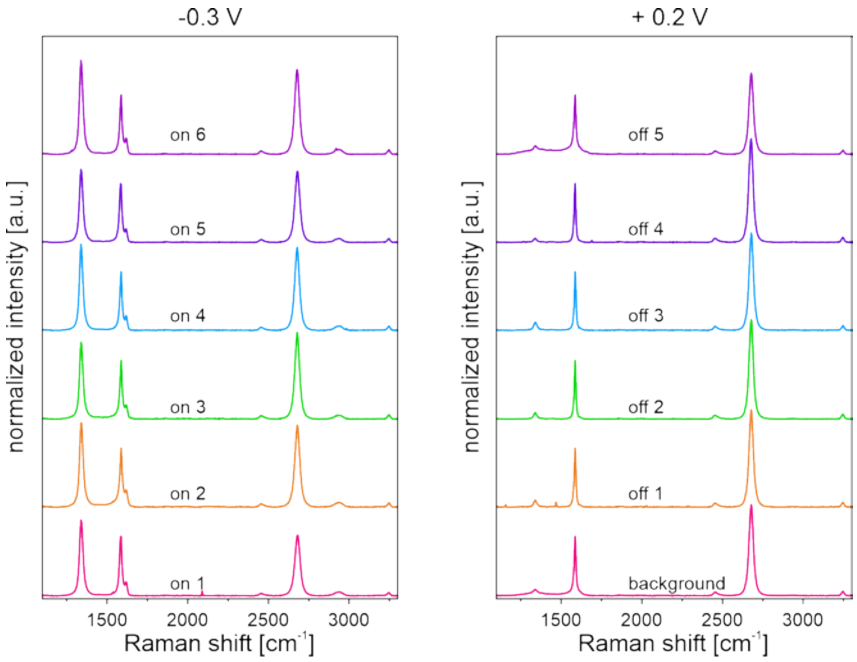

Figure S11: Normalized mean Raman spectra of all areas functionalized by laser 'writing' under the applied electrochemical potentials for 'on' and 'off'.

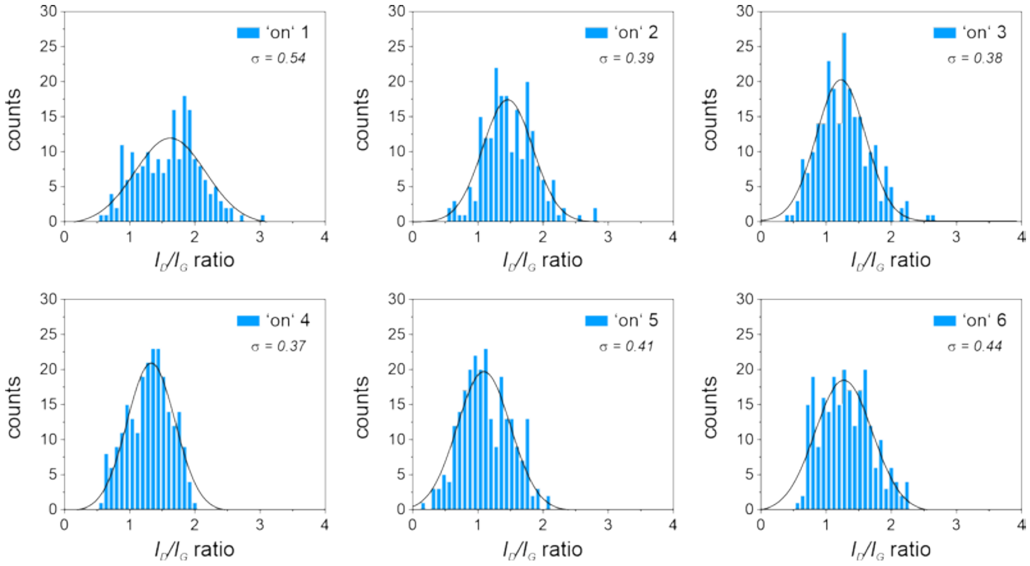

Figure S12: Histograms of the I_D/I_G ratios of the six functionalized graphene areas in Figure 6 under 'on' conditions using the same laser 'writing' parameters at an applied electrochemical potential of −0.3 V vs. ocp. Sigma values were obtained by Gauss fitting.

3.4. *In-Situ* 'Readout'

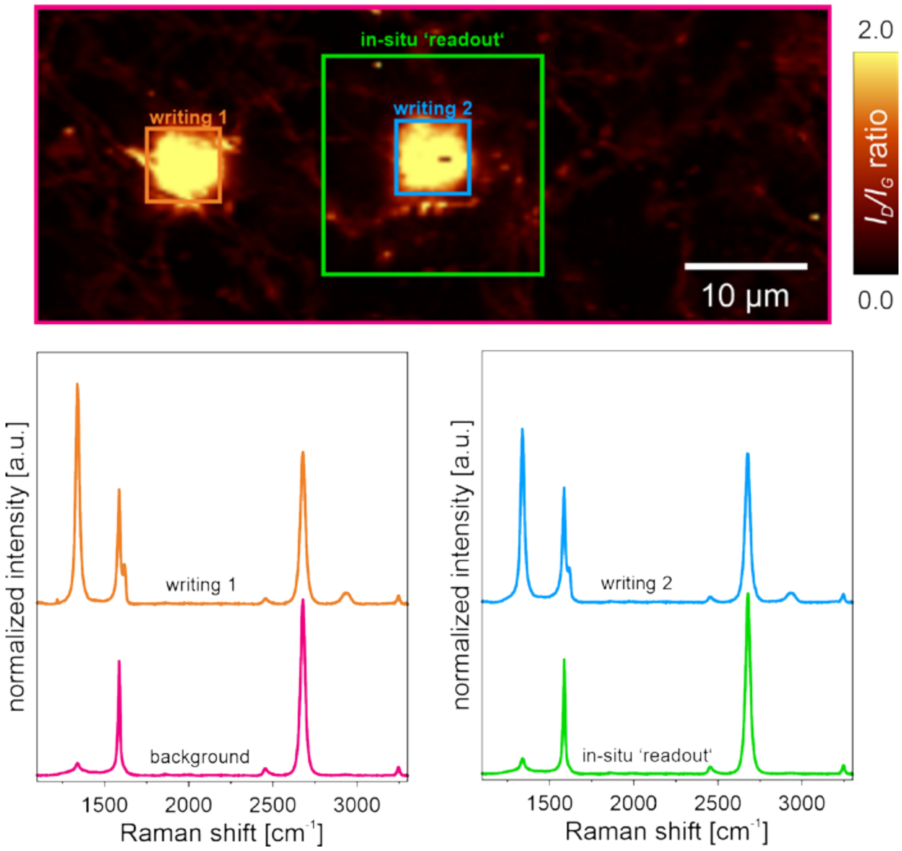

Figure S13: *Ex-situ* Raman I_D/I_G map (top) of the *in-situ* readout area and its surroundings, and the corresponding normalized mean Raman spectra (bottom). Writing 1 (orange) and writing 2 (blue) were functionalized at −0.3 V vs ocp ('on' potential). *In-situ* 'readout' (green) was performed at +0.2 V vs. ocp ('off' potential). The large *ex-situ* map (pink) was obtained on dry graphene after removing the reactant molecule.
Writing parameters: λ = 514 nm, P_L = 0.482 mW, t = 1 s, 5 x 5 µm, 10 x 10 points.
Reading parameters: λ = 532 nm, P_L = 5 mW, t = 1 s, 0.33 µm point distance.

4. References

[1] P. Giannozzi, S. Baroni, N. Bonini, M. Calandra, R. Car, C. Cavazzoni, D. Ceresoli, G. L. Chiarotti, M. Cococcioni, I. Dabo et al., QUANTUM ESPRESSO: a modular and open-source software project for quantum simulations of materials, *J. Phys.: Condens. Matter* **2009**, *21*, 395502.

[2] P. Giannozzi, O. Andreussi, T. Brumme, O. Bunau, M. Buongiorno Nardelli, M. Calandra, R. Car, C. Cavazzoni, D. Ceresoli, M. Cococcioni et al., Advanced capabilities for materials modelling with Quantum ESPRESSO, *J. Phys.: Condens. Matter* **2017**, *29*, 465901.

[3] M. F. Shojaei, J. E. Pask, A. J. Medford, P. Suryanarayana, Soft and transferable pseudopotentials from multi-objective optimization, *Comput. Phys. Commun.* **2023**, *283*, 108594.

[4] J. P. Perdew, K. Burke, M. Ernzerhof, Generalized Gradient Approximation Made Simple, *Phys. Rev. Lett.* **1996**, *77*, 3865.

[5] S. Ghosh, S. K. Behera, A. Mishra, C. S. Casari, K. K. Ostrikov, Quantum Capacitance of Two-Dimensional-Material-Based Supercapacitor Electrodes, *Energy Fuels* **2023**, *37*, 17836.

[6] C. Zhan, J. Neal, J. Wu, D. Jiang, Quantum Effects on the Capacitance of Graphene-Based Electrodes, *J. Phys. Chem. C* **2015**, *119*, 22297.

[7] G. Abbas, F. J. Sonia, M. Jindra, J. Červenka, M. Kalbáč, O. Frank, M. Velický, Electrostatic Gating of Monolayer Graphene by Concentrated Aqueous Electrolytes, *J. Phys. Chem. Lett.* **2023**, *14*, 4281.

[8] M. Massicotte, G. Soavi, A. Principi, K.-J. Tielrooij, Hot carriers in graphene - fundamentals and applications, *Nanoscale* **2021**, *13*, 8376.

[9] Y. Chen, Y. Li, Y. Zhao, H. Zhou, H. Zhu, Highly efficient hot electron harvesting from graphene before electron-hole thermalization, *Sci. Adv.*, *5*, eaax9958.
